# Assessing Immune Fitness in Oncological Rehabilitation—Validity and Responsiveness of the Immune Status Questionnaire and Single-Item Scale

**DOI:** 10.3390/curroncol33070415

**Published:** 2026-07-10

**Authors:** Anne M. S. de Hoop, Johanna A. Eggink, Cindy Veenhof, Cyrille A. M. Krul, Jelle P. Ruurda, Raymond H. H. Pieters, Karin Valkenet

**Affiliations:** 1 Research Group Innovation of Human Movement Care, Research Center for Healthy and Sustainable Living, HU University of Applied Sciences Utrecht, 3583CS Utrecht, The Netherlandsk.valkenet@umcutrecht.nl (K.V.); 2 Research Group Innovative Testing in Life Sciences and Chemistry, Research Center for Healthy and Sustainable Living, HU University of Applied Sciences Utrecht, 3583CS Utrecht, The Netherlands; 3 Department of Rehabilitation, Physiotherapy Science & Sport, University Medical Center Utrecht, Utrecht University, 3584CX Utrecht, The Netherlands; 4 Physiotherapy Science, Program in Clinical Health Sciences, University Medical Center Utrecht, Utrecht University, 3584CG Utrecht, The Netherlands; 5 Department of Surgery, University Medical Center Utrecht, Utrecht University, 3584CX Utrecht, The Netherlands; 6 Institute for Risk Assessment Sciences (IRAS)-Toxicology, Population Health Sciences, Faculty of Veterinary Sciences, Utrecht University, 3584CM Utrecht, The Netherlands

**Keywords:** immune fitness, immune system, physical therapy, cancer

## Abstract

Background: Immune fitness (IF) reflects the body’s ability to mount appropriate immune responses. Monitoring IF could improve tailored treatment in oncological rehabilitation. The Immune Status Questionnaire (ISQ) and the Single-Item Scale (SIS) were developed to assess IF, but their clinimetric properties in cancer rehabilitation remain unknown. Aims: To evaluate the construct validity, responsiveness, and correlation between the ISQ and the SIS in oncological rehabilitation. Methods: The study population included people participating in oncological rehabilitation during or within one year after medical treatment. Data were collected prospectively via questionnaires. Construct validity and responsiveness were assessed through predefined hypotheses, including correlations with fatigue, sleep problems, malnutrition risk, activity impairment, and physical functioning. Results: In total, 97 individuals were included in the analyses. Median ISQ and SIS scores were 8/10 and 7/10, respectively. Correlations ranged from r = −0.21 to r = −0.50. Only the SIS correlations with fatigue and physical functioning, and the ISQ correlation with fatigue, met the predefined thresholds. Responsiveness hypotheses were not confirmed. Conclusions: The ISQ and the SIS demonstrated low construct validity and responsiveness in this population. IF scores were higher than expected. Correlations showed links between fatigue, physical functioning, and IF. Future research should develop tools tailored to the complex immune disturbances experienced by cancer survivors.

## Simple Summary

Immune fitness describes how well the body can respond to health challenges and maintain a balanced immune system. Assessing immune fitness may help healthcare professionals to better tailor rehabilitation programs for people recovering from cancer. In this study, we evaluated two brief questionnaires that measure immune fitness in individuals participating in cancer rehabilitation during or shortly after treatment. The questionnaires showed some meaningful associations with fatigue and physical functioning, which suggests that perceived immune fitness may be linked to important aspects of recovery. However, associations with other health measures were weaker than expected, and the questionnaires were not able to adequately detect changes over time. Although participants generally reported relatively high levels of immune fitness, these findings indicate that these two questionnaires may not fully reflect the complex immune-related challenges experienced by cancer survivors. Future research should focus on developing more appropriate tools for this population.

## 1. Introduction

Every year, more than 120,000 individuals in the Netherlands are diagnosed with cancer. Many of them engage in training with an oncology physiotherapist before, during or after treatment [1,2]. People with cancer are a complex target group, as their workload capacity can fluctuate considerably over time due to the disease, the side effects of treatment, and many other personal factors [1,2]. One common side effect of cancer treatment is immunosuppression, which increases susceptibility to infectious diseases and reduces workload capacity [3,4,5,6]. Literature indicates that physical training can positively impact the immune system, although excessive workloads may have detrimental effects [7,8]. Therefore, monitoring immune functioning could enable the optimization of workload during oncological rehabilitation.

Currently, no consensus exists on the optimal measure for providing a comprehensive overview of immune functioning. Various blood markers can indicate the presence and functionality of immune cells and signaling molecules involved in immune functioning [9]. However, individual blood markers alone do not offer a complete picture of resilience against immunological stressors because they each reflect only a very small part of the system [9]. Second, they do not include the patient’s perception. Moreover, interpreting biomarkers can be challenging for physiotherapists, and invasive techniques are not feasible in their daily practice.

A potentially more relevant outcome measure for physiotherapy is immune fitness (IF) [10,11]. IF can be conceptualized as a subjective construct reflecting an individual’s perceived ability to adequately respond to health challenges, such as infections or physiological stressors [10,11]. In this context, IF is closely related to concepts such as resilience and perceived health status. In previous studies, immune fitness has been associated with susceptibility to illness (i.e., the number and severity of COVID-19 symptoms during the COVID-19 pandemic) [12], poorer physical and mental health [13] and quality of life [14]. By focusing on the individual’s experience of immune functioning, IF provides insight into how people perceive their capacity to maintain health when exposed to internal and external stressors. As such, IF can complement traditional biomarker-based assessment by providing a patient-centered perspective on immune functioning.

To date, two instruments have been specifically developed to assess IF: the Immune Status Questionnaire (ISQ) and the Immune Fitness Single-Item Scale (SIS) [10,11]. The ISQ retrospectively assesses immune-related symptoms experienced over a defined period, resulting in a score from 0 to 10, where higher scores indicate better IF [11]. The SIS is a scale ranging from 0 to 10 on which individuals rate their current immune fitness [10]. Both instruments have demonstrated acceptable validity and reliability in healthy populations and several clinical groups [10,11,15]. However, their psychometric properties have not been established in individuals in oncological rehabilitation.

The primary aim of this study was to evaluate the construct validity of the ISQ and the SIS for patients in oncological rehabilitation. Additionally, we examined the responsiveness of the ISQ and the SIS, and the correlation between the ISQ and the SIS. Identifying a valid and responsive instrument for individuals with cancer would enable oncology physiotherapists to assess IF during exercise programs for screening and monitoring purposes, thereby facilitating more personalized treatment.

## 2. Materials and Methods

### 2.1. Design and Setting

In this observational study, data was collected prospectively at baseline (patient demographics), and at one (T1), two (T2) and three months (T3) after baseline (primary and secondary outcomes). Data was collected between March 2023 and July 2025.

### 2.2. Participants

Participants were eligible for inclusion if they (a) were receiving treatment from an oncology physiotherapist, (b) were receiving, or were within 1 year after the completion of medical treatment for cancer at the onset of this study, (c) were 18 years or older and (d) were able to read and write the Dutch language. Individuals were excluded from the study if they had cognitive impairments that could affect their ability to accurately complete the questionnaires. Participants were included in the primary analyses if they completed the baseline questionnaire and at least one follow-up questionnaire.

Individuals were screened for eligibility by oncology physiotherapists in 19 different primary care physiotherapy practices in the Netherlands. Eligible participants received written information about the study. Next, the informed consent procedure was performed by a member of the research team via phone or email. Participants gave either written or verbal informed consent before participation in the study.

This study aimed to include 100 participants, since the COSMIN guidelines for measurement properties prescribe a minimum of 100 participants for a high-quality study on construct validity [16].

### 2.3. Hypotheses

Due to the novelty of this construct, there is no gold standard to compare our outcomes with. Thus, construct validity and responsiveness were assessed via hypothesis testing. Hypotheses were established as follows: literature was searched for outcomes that are proven to be associated with IF [11,17,18,19,20,21,22]. Next, based on clinical reasoning and consultation with oncology physiotherapists, those outcomes were selected that were assumed most likely to reflect a part of the construct of IF, and were most relevant for people in oncological rehabilitation. This led to the following hypotheses:

For construct validity:

- IF is fairly correlated with sleep problems (R > |0.3|);
- IF is fairly correlated with activity impairments (R > |0.3|);
- IF is fairly correlated with fatigue (R > |0.3|);
- IF is fairly correlated with the risk for malnutrition (R > |0.3|);
- IF is fairly correlated with physical functioning (R > |0.3|).

For responsiveness:

- The change in IF (T3-T1) is fairly correlated with the change in sleep problems (R > |0.3|);
- The change in IF (T3-T1) is fairly correlated with the change in activity impairments (R > |0.3|);
- The change in IF (T3-T1) is fairly correlated with the change in fatigue (R > |0.3|);
- The change in IF (T3-T1) is fairly correlated with the change in physical functioning (R > |0.3|).

The minimum expected correlation between the primary outcomes and comparators was 0.3, since they do not assess the same construct but are related health outcomes [23,24]. The risk of malnutrition was not included in the hypotheses for responsiveness, since the period between assessments (2 months) was expected to be too short to show meaningful changes in this study sample.

Convergent validity and responsiveness were assessed for both the ISQ and the SIS. Construct validity and responsiveness were considered good when ≥75% of the hypotheses could be confirmed (≥4 hypotheses for construct validity, ≥3 hypotheses for responsiveness), moderate when 50–74% were confirmed (3 for construct validity, 2 for responsiveness) and low when less than 50% were confirmed (≤2 for construct validity, ≤1 for responsiveness) [25].

### 2.4. Data Collection

Data were collected via questionnaires sent by email. Participants unable to complete them digitally received paper versions. Participants were requested to complete the questionnaires within one week. The baseline questionnaire assessed age, height, weight, type of cancer, type and frequency of medical treatment, and comorbidities. Over the following three months, participants received a monthly questionnaire including (a) the ISQ and SIS [11], (b) the Multidimensional Fatigue Index (MFI-20) for fatigue [26], (c) the Jenkins Sleep Scale (JSS) for sleep problems [27], (d) Work Productivity and Activity Impairment–general health (WPAI-GH) questionnaire for activity impairment [28], (e) the Patient-Reported Outcomes Measurement Information System physical functioning (PROMIS-PF) for physical functioning [29] and (f) the Patient-Generated Subjective Global Assessment short-form (PG-SGA-sf) for malnutrition risk [30].

### 2.5. Immune Fitness

IF was assessed with the ISQ and the SIS. The ISQ (Appendix A) assesses the frequency of seven immune-related symptoms over the past two weeks. Although the original questionnaire used a one-year timeframe, the developers, Versprille et al. (2019) recommended adapting this based on the target population and assessment goals [11]. For this study, a two-week period was chosen after consultation with oncology physiotherapists, considering symptom variability in this population. Good validity and reliability of the ISQ were demonstrated in healthy populations, and the ISQ can differentiate between poor and normal health [11].

The SIS (Appendix B) asks participants to self-rate their current immune fitness. Test–retest reliability of this scale is high [15]. To the knowledge of the authors, the validity of this scale has not yet been established in people with cancer or similar populations. A previous study has shown that the SIS is significantly associated with the presence and severity of COVID-19 symptoms [31]. A similar single-item scale assessing perceived immune functioning was associated with irritable bowel syndrome symptoms [32] and sleep quality [22]. Both the ISQ and the SIS yield scores ranging from 0 to 10, with scores below 6 indicating reduced immune fitness [11].

### 2.6. Fatigue

The MFI-20 has been recommended to assess fatigue in people with cancer [33,34]. It is a 20-item questionnaire, assessing fatigue in both mental and physical domains. For this study, the total score was used. The questionnaire is valid, with good internal consistency (Cronbach’s alpha 0.84) and responsiveness [26].

### 2.7. Sleep Problems

The JSS is valid, reliable and appropriate for measuring sleep problems in patients with cancer [35,36]. It is a 4-item questionnaire with questions regarding trouble falling asleep, waking up during the night, and waking up feeling tired, based on a 5-point Likert scale. The JSS results in a score between 0 and 20 points, with a higher score indicating more sleep problems.

### 2.8. Activity Impairments

The WPAI:GH is a 6-item questionnaire, resulting in the percentages of experienced work impairments and total activity impairment [28]. When a participant does not have a paid job, only the percent activity impairment will be calculated. Since activity impairment was available for all participants, this outcome was used for the analysis. The validity, reliability and responsiveness of the WPAI have been demonstrated in different healthy and diseased adult populations [28,37,38,39,40,41].

### 2.9. Physical Functioning

The PROMIS-PF (short form version v2.0—Physical Function 8b) is an 8-item questionnaire assessing problems with physical tasks in daily life. The reliability, validity and sensitivity to change were demonstrated in healthy adults and people with cancer [29,42]. The questionnaire results in a raw score between 8 and 40, which was converted to a T-score via the conversion table [43]. A T-score above 50 indicates better than average physical functioning, while a score below 50 indicates lower than average.

### 2.10. Risk of Malnutrition

The PG-SGA questionnaire is a valid screening instrument to assess the risk of malnutrition in people with cancer [30,44]. The short form is the first part of the assessment and can be completed independently by the patient. The questionnaire results in a numerical score ranging from 0 (no problems) to 36 (worst problems).

### 2.11. Statistical Analysis

Statistical analyses were performed in SPSS (version 29.0). All data were checked for missing values. Next, continuous data were checked for normality and outliers before the primary analyses.

Construct validity was assessed based on data from T1. In case no data was available at T1, values from T2 were used. For responsiveness, two data points (T1 and T3) were needed. In case of missing data, Little’s MCAR test was performed. When data was missing at random, data was imputed via multiple imputation via chained equations with 5 iterations. Next, analyses were performed with pooled outcomes based on Rubin’s rule. Data were not imputed when a participant missed all data for that time point, since there would be too little information available for reliable imputation results.

For construct validity, Spearman’s rho correlation coefficients (r_s_) were calculated between the primary outcomes (ISQ and SIS) and comparators (risk of malnutrition, physical functioning, sleep, activity impairments, fatigue). Spearman’s rho was the most appropriate since the ISQ and SIS are at the interval level and thus do not follow a normal distribution. For responsiveness, Spearman’s rho correlation coefficients (r_s_) were calculated between the change in the ISQ and SIS, versus the change in comparators (T0 to T3).

## 3. Results

Initially, 127 eligible persons agreed to be contacted by the researcher. Of these, 105 participants were enrolled in the study and completed the baseline questionnaire. Seven participants did not complete one questionnaire but rejoined afterward (T1: *n* = 3, T2: *n* = 4). Five participants dropped out before T2, and eight participants dropped out before T3. Reasons for dropout or missing a measurement were “feeling too sick” (*n* = 1), “not willing to share data” (*n* = 1), “forgot to respond” (*n* = 3), “technical issues” (*n* = 2), “no time or headspace” (*n* = 6) or unknown reasons (no response to email and telephone; *n* = 17).

Eventually, 97 participants completed the follow-up questionnaires at least at one time point and were therefore included in the analyses for construct validity. This sample size is considered sufficient for an adequate-quality study according to the COSMIN guidelines [16].

There was no missing data in this dataset. Eighty-one participants were included in the analysis for responsiveness since they had completed the questionnaires on T1 and T3. One value was imputed for the ISQ at T3, prior to this analysis.

Participant characteristics are shown in Table 1. Age ranged from 35 to 82 years; the mean age was 57 years (sd = 12) and the majority was female (71%). Physiotherapists confirmed that this male/female ratio corresponded with their population in daily practice. Breast cancer was the most prevalent diagnosis (44.8%) and hormone therapy was the most reported treatment (28.6%). At the time of data collection, 30 participants (28.6%) were not receiving any form of medical treatment.

### 3.1. Construct Validity

Group results on the outcome measures, as well as the results regarding hypothesis testing for construct validity, are shown in Table 2. The median ISQ was 8 (IQR 3) and the median outcome on the SIS was 7 (IQR 2).

The ISQ showed a significant correlation with fatigue (r_s_ = 0.35, *p* < 0.05), indicating that higher fatigue levels were associated with lower immune fitness. Correlations with sleep problems (r_s_ = −0.25, *p* < 0.05), activity impairments (r_s_ = −0.23, *p* < 0.05), risk for malnutrition (r_s_ = −0.21, *p* < 0.05) and physical functioning (r_s_ = 0.27, *p* < 0.05) were significant but with r_s_ < |0.3|. Because only one hypothesis was confirmed, the construct validity of the ISQ was low.

The SIS showed significant correlations with physical functioning (r_s_ = 0.40, *p* < 0.001) and fatigue (r_s_ = −0.50, *p* < 0.001). Higher physical functioning was associated with higher immune fitness, and higher fatigue levels were associated with lower immune fitness. Correlations with sleep problems (r_s_ = −0.23, *p* < 0.05), activity impairments (r_s_ = −0.23, *p* < 0.05) and risk for malnutrition (r_s_ = −0.24, *p* < 0.05) were significant but with r_s_ < |0.30|. Because two hypotheses were confirmed, construct validity of the SIS was low.

### 3.2. Responsiveness

Outcomes for responsiveness testing are shown in Table 3. All correlations for both the change in SIS and change in ISQ were ≤0.30. Similar to the cross-sectional analyses, the highest correlations were found for fatigue (r_s_ = −0.29, *p* < 0.05) and physical functioning (r_s_ = 0.24, *p* < 0.05), with the SIS. All hypotheses for responsiveness testing were rejected; thus, responsiveness of both the SIS and ISQ was low.

### 3.3. Changes in IF over Time

**Figure 1:**
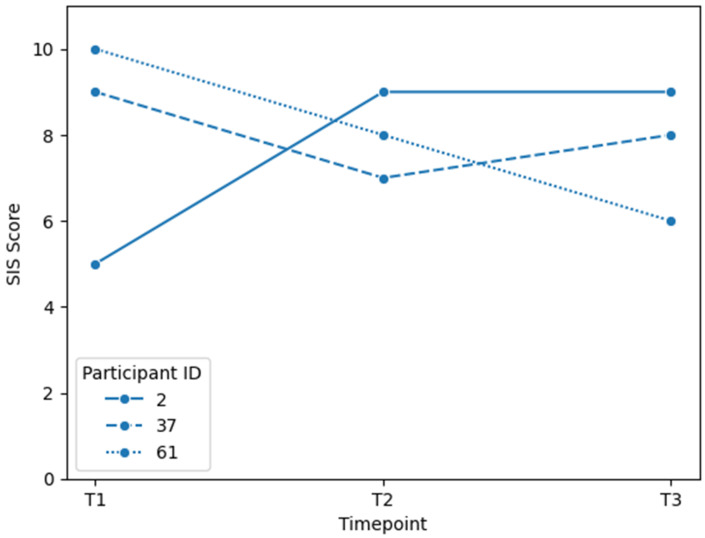
Examples of Single-Item Scale scores over time. Note. Each line represents one participant. SIS = single-item scale for immune fitness. The examples show that different patterns were observed over time.

**Figure 2:**
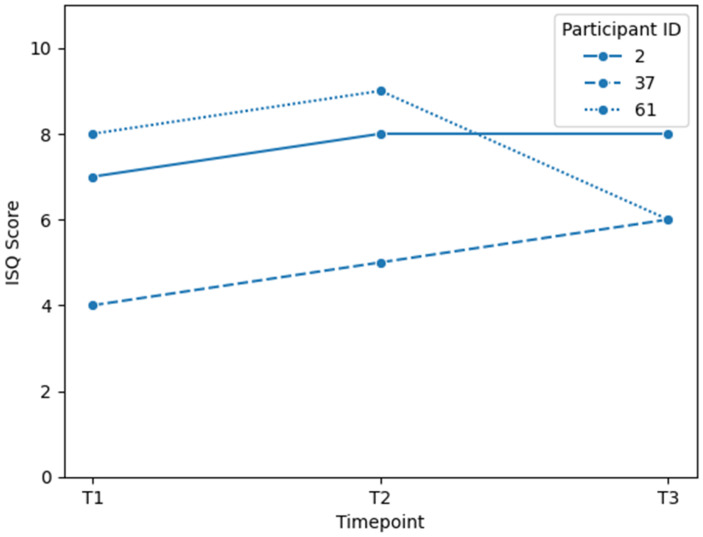
Examples of Immune Status Questionnaire scores over time. Note. Each line represents one participant. ISQ = Immune Status Questionnaire. The examples show that different patterns are observed over time.

Over time, changes were observed in both the ISQ and SIS, in both negative and positive directions. Examples of SIS and ISQ scores over time are shown in Figure 1 and Figure 2 respectively. Changes over time in all outcomes are more specifically described in Appendix C.

## 4. Discussion

This study evaluated the construct validity and responsiveness of the ISQ and the SIS in assessing immune fitness (IF) in oncological rehabilitation. Moderate correlations were found between the SIS and fatigue (r = −0.50) and physical functioning (r = 0.40) as well as a fair correlation between fatigue and the ISQ (r = −0.35). Less than 50% of the hypotheses were confirmed; therefore, the construct validity and responsiveness of both the ISQ and the SIS were low.

Consistent with previous research in other populations, this study confirmed a relationship between IF and fatigue [11,20]. Notably, the correlations between fatigue and both the ISQ (r = 0.35, *p* < 0.05) and the SIS (r = 0.50, *p* < 0.05), were stronger than the correlation observed between fatigue and the SIS in a previous study with a sample of healthy young adults (r = 0.15, *p* < 0.05) [11]. In addition to fatigue, our study identified a moderate correlation between the SIS, and physical functioning (r = 0.40, *p* < 0.05). To the authors’ knowledge, this relationship has not been established in earlier research. Significant correlations were also found with sleep problems, activity impairments and the risk of malnutrition, for both the SIS and the ISQ. Although these coefficients were below the predefined cut-off point of 0.3, they were all above 0.2, which confirms a small relationship with IF.

The longitudinal analysis identified significant correlations between an increased SIS with decreased fatigue (r = −0.29, *p* < 0.05) and increased physical functioning (r = 0.24, *p* < 0.05). Although the correlations were below the predefined threshold to confirm hypotheses for responsiveness, they are relevant to explore further in future research. Other correlations were not significant and r ≥ 0.2. Possibly, the changes in sleep problems, physical fitness, activity impairments and fatigue in this population are too complex to be associated with the ISQ. Considering the complexity of changes in symptoms related to immune functioning and immune fitness in this population, future research should explore this longitudinal analysis based on different timeframes.

In an earlier qualitative study, oncology physiotherapists linked impaired immune functioning to reduced physical fitness, lower loading capacity, slower recovery and increased fatigue [17]. The assessed associations between IF and physical functioning and fatigue are in line with this, since IF can be viewed as a derivative of immune functioning. Given that both physical functioning and fatigue are key outcomes in oncology physiotherapy, the observed associations with IF underscore the potential value of integrating the outcome of IF into clinical reasoning in oncological rehabilitation. An aspect that should be explored in future research is the relationship between IF and the different dimensions of fatigue. This study focused on the total outcome of the multidimensional fatigue inventory questionnaire, while physical and mental fatigue may have a different relationship with IF.

Interestingly, the median ISQ and SIS scores were higher than anticipated, with scores of 8 (IQR 3) and 7 (IQR 2) out of 10 respectively. Given that immunosuppression is common during and after cancer treatment and that immune recovery can take months [3,6], a lower median IF was expected. For comparison, a similar mean score for immune fitness (7) was found in two other study samples from the general population [18,45]. Our relatively high scores could be explained by the multifactorial nature of IF: IF is a subjective outcome, which is not only based on symptom frequency, but also influenced by the type, severity and impact of complaints, as well as coping mechanisms [10]. It is plausible that participants who seek support from a physiotherapist possess relatively strong coping mechanisms for health-related complaints. Additionally, participants may perceive their complaints as less severe compared to their previous experiences because they have been through a severe disease and heavy treatment. Together this could explain the relatively high IF that was assessed in the current study.

Based on the results of this study, the construct validity and responsiveness of the ISQ and the SIS in oncological rehabilitation are low. This suggests that these measures do not adequately capture IF in this population. One possible explanation is that people with cancer and cancer survivors often experience complex, long-lasting and heterogeneous immune disturbances. These disturbances may not be sufficiently represented by the ISQ and the SIS, which were not specifically designed for this population. A more tailored approach may involve a multidimensional questionnaire that accounts for the diversity, severity and temporal variability of immune-related symptoms. It could also integrate domains such as fatigue, and psychological factors such as stress and coping, which are particularly relevant in oncology. Lastly, perhaps the questionnaire should be tailored to subgroups of patients, for example, those receiving specific types of medical treatment. Further research is needed to determine which domains are essential for assessing IF during and after cancer treatment.

IF remains a relevant concept in oncology physiotherapy. Unlike biomarkers, which reflect isolated immune mechanisms, IF provides a holistic perspective, capturing the overall adaptability of the immune system to stressors as well as how individuals perceive their immune health. However, the conceptualization of IF is still evolving, and inconsistencies in terminology persist. In the literature, IF has been described both as “adequate functioning of the immune system” as well as the “perceived functioning of the immune system” [10,11]. The symptom-based assessment of the ISQ aligns more closely with the former, while the SIS better captures the perception of IF. In oncological rehabilitation, *perceived* IF may be particularly relevant. Patients’ perceptions influence how they interpret symptoms, make health-related decisions, and engage with rehabilitation. Understanding these perceptions can provide valuable insights into resilience and recovery.

The accuracy of subjective assessments such as the SIS may be affected by education and literacy. IF is a complex concept, and patients require a basic understanding to assess it meaningfully. Although the SIS includes a definition, comprehension may vary. Moreover, the current format may be challenging for individuals with low literacy, potentially limiting its reliability and accessibility. Possibly, educating patients on the concept of IF is an essential part of integrating this measure into the rehabilitation trajectory. Future research should aim to improve the clarity and accessibility of the SIS, ensuring the measure is comprehensible across varying educational backgrounds.

## 5. Strength and Limitations

A strength of this study is that the sample closely reflected the target population seen in physiotherapy practice, including patients with various types of cancer, at different stages of treatment, and undergoing diverse therapeutic approaches. This enhances the generalizability of the findings to the target population. Additionally, the sample size met the recommended standards set by the COSMIN guidelines [16]. The heterogeneity in the study population on the other hand, could have influenced the results. It is possible that the relationship between the comparators and immune fitness is slightly different for patients in different treatment trajectories or with different timing since treatment. Therefore, future research should explore the clinimetric properties of the ISQ and the SIS for different subpopulations of people with cancer.

Another potential limitation of this study is the relatively short two-week timeframe used for the retrospective assessment of the ISQ. This duration was selected in consultation with oncology physiotherapists, aiming to find balance between capturing meaningful changes in immune-related complaints while also providing a reliable overall estimate of immune fitness. The timeframe may influence the outcomes: if the timeframe is too short, certain patterns—such as those related to medical treatment schedules—may not be adequately accounted for. Conversely, if the timeframe is too long, relevant fluctuations in immune fitness could be missed. More research is needed to determine the optimal retrospective period for assessing IF with the ISQ for this population.

Another limitation stems from the limited knowledge of factors related to IF, particularly in individuals with cancer. As a result, it remains challenging to formulate definitive hypotheses for construct validity and responsiveness testing. In this study we have combined knowledge from the existing literature, clinical reasoning and expert opinion to identify variables most likely to correlate with IF. Nevertheless, it is possible that alternative variables might have provided a better fit. For instance, coping was not included in the hypotheses for construct validity and responsiveness, although in recent research—published after the finalization of the current research protocol—it was suggested that coping may influence immune fitness [10]. Future research should explore a broader range of psychosocial and physiological factors to refine the conceptual framework of immune fitness. In such future work, multivariable analyses would offer additional insight by clarifying whether observed associations are independent or influenced by other participant characteristics.

Lastly, the use of self-reported questionnaires may introduce response and recall bias, and the use of multiple administration modes may contribute to bias. Several measures were implemented to mitigate these effects. Questionnaires were administered sequentially without access to prior responses, and participants were not informed in advance about repeated measurements of the same questionnaires, thereby reducing consistency-driven answering. Additionally, the recall period of the questionnaires that were used for responsiveness analyses lay within the period since the previous assessment (one month) and was explicitly stated on the questionnaire. Lastly, standardized instructions, invitations and reminders were used to ensure consistency in data collection. Despite these precautions, some degree of bias cannot be excluded.

## 6. Conclusions

In conclusion, this study could not confirm the construct validity and responsiveness of the Immune Status Questionnaire and the Single-Item Scale for immune fitness in the oncological rehabilitation setting. The cross-sectional analysis indicated significant correlations with all outcomes, but only the associations with physical fitness and fatigue were above the predefined threshold. Future research should further explore the factors associated with IF. Furthermore, the SIS and the ISQ might be tailored for this specific population to better assess the unique immune challenges faced by people with cancer and cancer survivors. Lastly, attention should be given to the evolving definition of IF, and to improving the clarity of the questionnaires to ensure reliable use across diverse educational and literacy levels. The development of a tailored tool to assess IF for oncological rehabilitation could support personalized training programs.

## Figures and Tables

**Table 1:** Participant baseline characteristics (*n* = 97).

Variable Name			
Age, mean (sd)		57	(12)
Sex, *n* (%)	Female	69	(71)
	Male	28	(29)
BMI, mean (sd)		26.5	(4.5)
Type of cancer, *n* (%)	Breast	45	(46.4)
	Reproductive organs	13	(13.4)
	Gastro-intestinal	10	(10.3)
	Lung	8	(8.25)
	Bone marrow	4	(4.1)
	Lymphatic	4	(4.1)
	Multiple types simultaneously	2	(2.1)
	Other	8	(8.3)
	Unknown	2	(2.1)
Treatment status at baseline ^1^, *n* (%)	Chemotherapy	21	(21.7)
	Radiotherapy	5	(5.2)
	Hormone therapy	30	(39.9)
	Immune therapy	18	(18.6)
	No current treatment	26	(26.8)
Comorbidities ^1^	Cardiovascular	12	(12.4)
	Osteoarthritis	8	(8.2)
	Neurological	7	(7.2)
	Lung	6	(6.2)
	Diabetes	4	(4.1)
	Rheumatic	1	(1.0)
	Gastrointestinal	1	(1.0)
	Other	15	(15.5)

Note. *n* = number of participants, sd = standard deviation. ^1^ Total *n* exceeds 100 because some participants received multiple treatments simultaneously/had multiple comorbidities.

**Table 2:** Outcomes for construct validity testing.

	Median (IQR)		r_s_ with ISQ (95%CI)		r_s_ with SIS (95% CI)	
*Primary outcomes*						
Immune fitness (ISQ)	8	(3.0)			0.40 *	(0.21:0.57)
Immune fitness (SIS)	7	(2.0)				
*Variables for hypotheses testing for construct validity*						
Sleep problems	11	(8.0)	−0.25 *	(−0.42:−0.05)	−0.23 *	(−0.42:−0.21)
Activity impairments	60.0	(40.0)	−0.23 *	(−0.41:−0.03)	−0.23 *	(−0.42:−0.02)
Fatigue	61.0	(21.0)	−0.35 *	(−0.52:−0.17)	−0.50 *	(−0.64:−0.31)
Risk for malnutrition	3	(5.0)	−0.21 *	(−0.38:−0.03)	−0.24 *	(−0.24:−0.04)
Physical functioning	40.8	(7.8)	0.27 *	(0.08:0.46)	0.40 *	(0.22:−0.55)

Note. * *p* < 0.05, r_s_ = Spearman’s rho, ISQ = Immune Status Questionnaire, SIS = ISQ single-item scale, IQR = interquartile range. Shaded cells indicate that the hypothesis (r_s_ ≥ |0.30|) was confirmed.

**Table 3:** Outcomes for responsiveness testing.

	r_s_ with Change ^1^ in ISQ (95% CI)		r_s_ with Change ^1^ in SIS (95% CI)	
*Primary outcomes*				
Change ^1^ in ISQ-MIS			0.23 *	(0.00:0.43)
Change ^1^ in ISQ-SIS				
*Variables for hypotheses testing for responsiveness*				
Change ^1^ in sleep problems	−0.06	(−0.29:0.17)	−0.16	(−0.37:0.07)
Change ^1^ in activity impairments	−0.10	(−0.32:0.13)	−0.02	(−0.24:0.21)
Change ^1^ in fatigue	−0.17	(−0.39:0.05)	−0.29 *	(−0.49:−0.07)
Change ^1^ in physical functioning	0.20	(−0.02:0.41)	0.24 *	(0.02:0.44)

Note. ^1^: Change between T1–T3 * *p* < 0.05, r_s_ = Spearman’s rho, ISQ = Immune Status Questionnaire, SIS = single-item scale. All hypotheses were rejected because all correlation coefficients were below the predefined threshold (r_s_ ≥ |0.30|).

## Data Availability

The original data presented in the study are available in DataverseNL at https://doi.org/10.34894/UYFH5L.

## Appendix A. Immune Status Questionnaire

### Immune Status Questionnaire

1. **Please indicate how often you have had the following complaints in the past two weeks:**

	**Never**	**Sometimes**	**Regularly**	**Often**	**(Almost) Always**
Sudden high fever					
Diarrhea					
Headache					
Skin problems (e.g., acne and eczema)					
Muscle and joint pain					
Common cold					
Coughing					

## Appendix B. Single-Item Scale

2. **Rate your immune fitness**

Immune fitness refers to the capacity of the body to respond to health challenges (such as infections) by activating an appropriate immune response, essential to maintain health, prevent and resolve disease, and improve quality of life.

At this moment, I rate my immune fitness as follows:

**0**	**1**	**2**	**3**	**4**	**5**	**6**	**7**	**8**	**9**	**10**
Very Poor										Excellent

## Appendix C. Change Scores

	**T1–T2**				**T2–T3**			
	**↑/→**		**↓**		**↑/→**		**↓**	
Immune fitness (SIS)	+1.00	(1.00)	−1.00	(1.00)	+0.00	(1.00)	−1.00	(1.00)
Immune fitness (ISQ)	+1.00	(2.00)	−1.00	(1.00)	+1.00	(1.00)	−1.50	(1.00)
Sleep problems	+1.00	(3.00)	−3.00	(3.50)	+2.00	(2.00)	−2.00	(3.00)
Activity impairments	+10.00	(20.0)	−20.00	(20.00)	+0.00	(20.00)	−20.00	(20.00)
Fatigue	+3.00	(6.00)	−5.00	(5.75)	+5.00	(5.50)	−7.50	(6.00)
Risk for malnutrition	+0.00	(2.00)	−2.00	(3.00)	+0.00	(3.00)	−2.00	(4.25)
Physical functioning	+1.30	(1.75)	−2.55	(3.30)	+2.25	(4.96)	−2.10	(2.65)
Note. Change scores expressed in median (inter quartile range). SIS = single-item scale, ISQ = immune status questionnaire. ↑/→; median change in participants who increased or remained stable, ↓: median change in participants who decreased.								
